# Cost‐Effectiveness of 2 Support Models for a Healthy School Initiative*

**DOI:** 10.1111/josh.12931

**Published:** 2020-07-21

**Authors:** Matthew P. Finster, Jill Feldman

**Affiliations:** ^1^ Senior Study Director, (matthewfinster@westat.com), Westat, Inc., 1600 Research Blvd., Rockville, MD 20850; ^2^ Senior Study Director, (jillfeldman@westat.com), Westat, Inc.,1600 Research Blvd., Rockville, MD 20850

**Keywords:** organization and administration of school health programs, cost‐effectiveness, healthy eating, childhood obesity, physical activity, elementary schools

## Abstract

**BACKGROUND:**

In school year (SY) 2014‐2015, 128 schools in 24 districts and 14 states were randomly assigned to receive either onsite or online support to implement a school‐based wellness program. The objective of this study was to assess the cost‐effectiveness (CE) of the 2 models of implementation support: onsite and online.

**METHODS:**

We adapted the “ingredients method” for the CE analysis. Using expenditure data, we tabulated the costs of relevant expense categories and allocated the appropriate proportion to determine the total costs of providing each type of support for 4 years (SY 2014‐2015 through SY 2017‐2018). We divided the average cost per school by the average change in school wellness policies and practices, using assessment data provided by the program provider, to calculate a CE ratio for schools in each group.

**RESULTS:**

Findings indicate that when the program is implemented as intended, online support is, on average, approximately 1.3 times more cost‐effective than onsite support at the end of 4 years.

**CONCLUSIONS:**

By demonstrating the relative CE of 2 approaches to supporting the implementation of a school health program, this study provides further insight on more efficient interventions for improving overall school wellness.

Obesity is a persistent public health concern. Childhood and adolescent obesity rates remain relatively high despite efforts to reduce them.^1^, ^2^ The obesity epidemic in this country is well‐documented, with over one‐third of children and adolescents classified as being overweight (15%) or obese (19%),^3^ and disparities among racial and ethnic groups remain a particular concern.^2^ In addition to adverse health effects, overweight and obesity in childhood and adolescence is related to negative school outcomes such as decreased executive functioning skills;^4^ increased rates of detention, absenteeism, and tardiness;^4^ and negative social‐behavior outcomes.^5^ Not surprisingly, schools have been identified as a key setting to address childhood and adolescent obesity,^6^, ^7^ and numerous school‐based health programs have been developed, implemented, and evaluated across the United States.^8^ It is clear that healthy habits are formed in childhood, and research indicates that schools can play an important role in improving student weight outcomes and dietary behavior.^9^, ^10^, ^11^, ^12^ Randomized trials of school‐based health interventions have demonstrated reductions in children's weight.^13^, ^14^ In addition, there is some evidence of a clear dose‐response relationship between exposure to/duration of school‐based health programs and improvements in students' weight status.^15^ Whereas research has shown that school programs can effectively promote healthy eating and physical activity among children and adolescents,^16^, ^17^ and cost‐effectiveness (CE) research has demonstrated that school‐based obesity prevention programs and physical activity interventions are relatively cost‐effective,^18^, ^19^ there remains limited, timely information on the CE of alternative approaches for supporting the implementation of school health programs.^20^ The objective of this study was to evaluate the CE of 2 types of implementation support, onsite and online, for a school‐based health program and to identify which alternative is more cost‐effective.

## SCHOOL‐BASED HEALTH INTERVENTION

Recognizing the potential for schools to provide more healthful nutrition and physical activity environments for students, a wellness‐focused organization created a school‐based health program to support schools with students of low socioeconomic status (SES) to create environments in which physical activity and healthy eating are encouraged and accessible before, during, and after school. The program supports schools' implementation of health‐focused policies and practices via a model that provides either onsite support in the form of training and technical assistance (TA) provided by a program manager and team of content advisors, or online support in the form of self‐guided online program participation and access to resources. Program managers and call center staff guide onsite and online schools as they implement the program. The program's goals are to support and recognize schools that establish a healthy school environment as an education priority; provide healthier food options for students during the regular and extended school day; increase opportunities for students to exercise and play before, during, and after school; and develop programs to help teachers and staff become healthy role models. The program provides schools in participating districts with resources and TA to build their capacity to implement a 6‐step process and best practice framework designed to integrate school wellness efforts. The 6‐step process is repeated annually to expand and institutionalize wellness policies and practices, with implementation support provided online or onsite.

**Figure 1:**
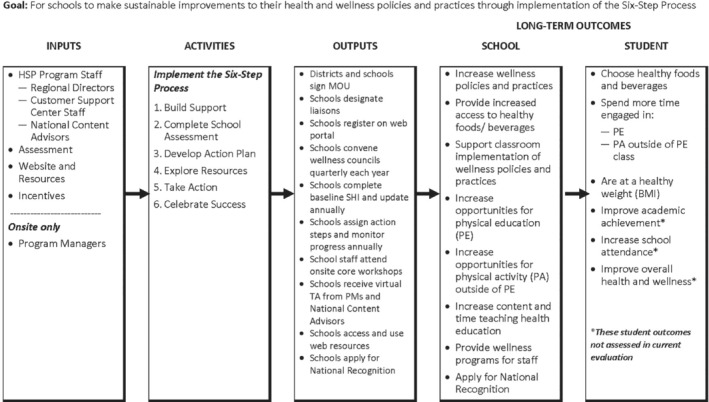
School Health Program Logic Model. Note. BMI, body mass index; MOU, memorandum of understanding; PM, project manager; SHI, School Health Index; TA, technical assistance

Schools receiving onsite TA from program managers were encouraged to attend district training sessions and statewide summits and to apply for the National Recognition Program—an award indicating that the school meets standard health best practices. Also, for onsite schools, program managers arranged in‐person visits by national content experts, as requested. Online schools had access to similar support by email and telephone. Any school implementing the program had access to online resources, including toolkits, step‐by‐step instructions, and access to funding opportunities. The program logic model (Figure 1) includes details related to inputs, activities, outputs, and outcomes. Although in the original design, the 2 types of support (onsite and online) were meant to be distinct and maintained for the length of the study, over time, program managers often assisted online schools in addition to onsite schools. This fact has important implications for the costs of personnel allocated to each type of support, which are discussed further below.

## METHODS

To identify which type of support (onsite vs online) helps schools improve their overall wellness levels, as measured by changes in assessment data, for the lowest relative cost, we applied the “ingredients method”^21^ to monetize and compare the costs of increasing overall wellness associated with each type of implementation support. The ingredients method involves identifying and specifying the ingredients of each type of implementation support, valuing, and pricing ingredients, and calculating total costs. The cost calculations are paired with program impacts to perform a CE analysis, which is discussed in more detail below.

### Participants

Over the study period, 128 elementary schools serving students in low‐SES neighborhoods in 24 districts and 14 states were randomly assigned to receive onsite or online implementation support and began implementing the program. The cost data are based on the original sample of 128 schools (64 onsite, 64 online) and was used to generate an average cost per school for each type of support. Outcome data, consisting of baseline and Year 4 assessment scores, was available for 68 (43 onsite, 25 online) schools; these 68 schools are included in this analysis.

### Measuring Costs

To estimate the total costs of providing each type of support over 4 years, we obtained expenditure data for each program ingredient: personnel, facilities, equipment and materials, and other costs. Personnel included salaries of program managers, content experts, field operations specialists, marketing and communications staff, information technology staff, and call center support staff. We determined the costs of personnel for each type of support by prorating staff salaries based on conversations with the program provider about the number of schools served by staff, and then we adjusted the staff's full‐time equivalent (FTE) rates accordingly. For example, based on the typical number of schools served, 0.55 FTE of the average program manager's salary was ascribed to onsite support. Facility, equipment and materials, and other costs were ascribed in a similar manner. We used actual facility costs and equipment and materials costs associated with providing each type of support and prorated the costs based on the FTEs applied to their salary rates. “Other costs” refers to all other ingredients that do not fit readily into the categories above, including, for example, indirect costs, professional fees, travel, and meeting expenses.

For onsite support, we used the following FTEs: 0.55 FTE for program managers, 0.08 FTE for content experts, and 0.01‐0.02 for field operations specialists, marketing and communications staff, information technology staff, and call center staff. For online support, we used similar FTEs except for program managers, because, contrary to the intervention design, these staff also provided support to schools in the online group; for this reason, we attributed program manager FTEs of 0.00 (none) for year 1, 0.45 for year 2, and 0.52 for years 3 and 4 for online support.

### Measuring Program Effectiveness

To measure program effectiveness, we used differences in each school's assessment scores across the 4 years. Schools monitored their progress in implementing the program using a subset of items from the US Centers for Disease Control and Prevention's School Health Index (SHI).^22^ The items used in this study measure the extent to which schools implemented a range of health policies and practices within 6 core wellness areas:

1. School health policies and environment (14 items);
2. Health education (6 items);
3. Physical education and other physical activity programs (12 items);
4. Nutrition services (8 items);
5. Health promotion for staff (6 items); and
6. Family and community involvement (3 items).

Responses are provided using a 4‐point scale, where 4 = fully in place, 3 = partially in place, 2 = under development, and 1 = not in place. Assessment scores range from 0% to 100% of items fully implemented. In the first year, referred to as the baseline year, the average overall wellness score for schools in both groups was 61% of items fully implemented, 59% for onsite schools, and 63% for online schools. Baseline equivalence results are presented in Table 1.

**Table 1:** Baseline Equivalence Testing Results (SY 2014‐2015)

	Onsite (N = 43)		Online (N = 25)		Adjusted mean difference between groups		
School Characteristic	Mean	SD	Mean	SD	Mean	ES	p‐value
Assessment items complete at baseline	0.59	0.20	0.63	0.18	0.04	0.22	.39
Students receiving free or reduced price lunch	0.66	0.20	0.71	0.18	0.05	0.25	.33
Female students	0.50	0.03	0.49	0.02	−0.01	0.23	.37
School size	543.21	162.86	518.84	152.37	−24.37	0.16	.55

Note. Effect sizes (ES) within .05 and .25 standard deviations are considered equivalent. This analysis used assessment data from the provider's database and the US Department of Education's Common Core of Data.

### Cost‐Effectiveness Ratio

To conduct the CE analysis, we combined cost and effectiveness measures. For each type of support, we divided the average cost per school by the average change in wellness policies and practices from years 1 to 4. This calculation provides a CE ratio, interpreted as the cost per unit of effectiveness; that is, a 1 percentage point increase on the assessment, for each type of support. We followed these steps to conduct the CE analysis:

- Obtained data related to actual costs of personnel, facilities, materials and equipment, and other inputs, such as travel and overhead from program developers, to calculate the costs of providing online and onsite implementation support to the original 128 study schools (64 online, 64 onsite) during the 4 years of program implementation (SY 2014‐2015 through SY 2017‐2018).
- Tabulated the costs of relevant expense categories and allocated the appropriate proportion, to determine the total costs of providing each type of support for years 1, 2, 3, and 4 (SY 2014‐2015 through SY 2017‐2018).
- Divided the total cost of each type of support by the number of schools originally in each group (64 onsite, 64 online), to calculate the average cost of supporting a school using each type of support for all 4 years.
- Determined the CE metric using the average change in school wellness policies and practices (as measured by the assessment) from years 1 to 4, to measure the effectiveness of each type of support for the fourth year of program implementation, and created a matched sample based on fidelity, baseline and year 4 assessment scores, and school characteristics (onsite N = 43, online N = 25).
- Divided the average cost per school by the average change in school wellness policies and practices to calculate a CE ratio.

The resulting CE ratio provides information about how much it costs over 4 years to increase school wellness policies and practices by 1 percentage point on the assessment. The type of implementation support with the lower CE ratio is the less expensive approach for achieving a 1 percentage point increase in school wellness policies and practices.

## RESULTS

### Program Costs

Table 2 summarizes the costs of the major ingredients associated with providing 4 years of onsite and online implementation support. Providing onsite implementation support costs $336,176, or $5253 per school, most of which (68%) is attributable to personnel costs. Providing online support costs $241,433, or $3772 per school, with a similar proportion attributable to salaries.

**Table 2:** Costs of Providing Onsite and Online Implementation Support, SY 2014‐2015 (Y1) Through SY 2017‐2018 (Y4)

	Onsite Support (N = 64)			Online Support (N = 64)		
Costs by Category	Amount ($)	Amount per School ($)	Percent of Total	Amount ($)	Amount per School ($)	Percent of Total
Personnel	229,107	3580	68	163,489	2555	68
Facilities	5890	92	2	4086	64	2
Materials and equipment	6948	109	2	3902	61	2
Other inputs (overhead, etc.)	94,231	1472	28	69,956	1093	29
Total costs	336,176	5253	100	241,433	3772	100

Note. Differences may be due to rounding to nearest dollar. Sum exceeds 100% due to rounding.

### Program Effectiveness

**Figure 2:**
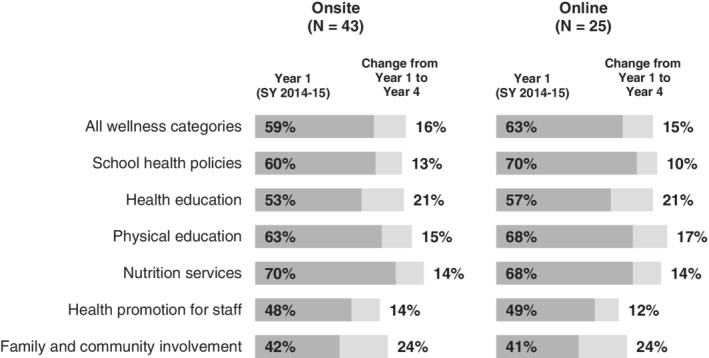
Change in Average Percentage of Items Fully Implemented at the End of 4 Years for Onsite and Online Support. Note. Differences in outcomes by assessment category between onsite and online schools are non‐significant (p > .05). This analysis used assessment data from the provider

Between onsite and online schools, there were no statistically significant differences in wellness gains after 4 years of program implementation. Across all wellness areas, onsite schools fully implemented more policies and practices in year 4 compared to baseline levels, rising 16 percentage points from 59% to 75%. Similarly, online schools fully implemented more policies and practices in year 4 compared to baseline levels, rising 15 percentage points from 63% to 78% (Figure 2).

### Cost‐Effectiveness Ratio

Table 3 presents the results of the CE analysis for the 4‐year period of SY 2014‐2015 through SY 2017‐2018. For onsite schools, the cumulative cost to increase overall wellness by 16 percentage points over 4 years was $5253. Based on these results, we calculated a CE ratio of $336, which indicates that, on average, it cost $336 to increase a school's overall wellness by 1 percentage point as measured by the assessment. In comparison, for online schools, the cumulative cost to increase overall wellness by 15 percentage points over 4 years was, on average, $3772, resulting in a CE ratio of $256.

**Table 3:** Cost‐effectiveness (CE) of Onsite and Online Implementation Support, SY 2014‐2015 (Year 1) through SY 2017‐2018 (Year 4)

	Type of Support	
CE Components	Onsite	Online
Costs: Average cumulative cost per school	$5253	$3772
Effectiveness: Average percentage point increase in overall wellness (SHI) score, years 1 through 4	16	15
CE ratio*	$336	$256

Note. Numbers were rounded to the nearest whole number.

SHI, School Health Index.

* The CE ratio indicates the average cost per school for an average increase of 1 percentage point, as measured by the assessment.

Although onsite support is slightly more effective (average percentage point increase in assessment score of 16 vs 15), it cost $1481 more per school to support an onsite school than an online school. The type of support that yields a given level of effectiveness for the lowest cost (or, conversely, the program that yields the highest level of effectiveness for a given cost) is more cost‐effective.^23^ Based on the CE ratios, it cost an average of $336 to increase an onsite school's overall wellness score by 1 percentage point on the assessment, compared to $256 for the same improvement in an online school. The CE ratios indicate that online support was, on average, approximately 1.3 times more cost‐effective than onsite support at the end of 4 years ($336 divided by $256).

Based on projections using the CE ratios, it would cost an average of $4007 per online school, versus the $5253 average cost per onsite school, to achieve a 16 percentage point increase in wellness outcomes after 4 years. We arrived at this estimate by multiplying the online school CE ratio of $256 by 16, which is the percentage point increase in wellness as measured by the assessment for onsite support. This estimate assumes that online schools with the current CE ratio of $256 would be able to achieve the same increase in wellness outcomes as the onsite schools did.

## DISCUSSION

Our results suggest that providing schools with online support to implement school health programs may be a more cost‐effective approach than providing onsite implementation support. It is worth noting that the costs of providing online support are mostly fixed and do not vary, regardless of the number of schools supported. Therefore, all else being equal, increasing the number of schools supported online would make online support even more cost‐effective than onsite support, because onsite support relies more on the variable and substantial costs associated with program managers' salaries.

Whereas studies have examined the overall CE of school health and activity programs,^18^, ^19^, ^24^ none that we found have examined the CE of different types of implementation support. This study contributes to the literature on school‐based health programs by demonstrating how program implementation can be effectively supported using online tools and call center staff, compared to the more time‐ and resource‐intensive onsite support often offered.

This study is subject to several limitations noted in similar CE research efforts. First, the CE ratios are highly dependent on the costs of one key ingredient: program manager salaries. Program managers were initially supposed to support onsite schools only. Based on the results of a related implementation study, which included annual interviews with program managers in addition to the time they logged supporting onsite and online schools, we adjusted the actual costs associated with those salaries to reflect a more realistic estimate of the time that program managers spent providing support to online schools. Over the course of the 4 years, we adjusted the costs of program manager salaries for online support by adding 0.45 FTE to the cumulative costs of ingredients in year 2 and by adding 0.52 FTE in years 3 and 4. If we had not adjusted the program managers' FTE across the 4 years for the schools receiving online support, the CE ratios would indicate that online support is closer to 3.4 times more cost‐effective, rather than 1.3 times more cost‐effective. This finding underscores the importance of using multiple sources of data to ascertain and ascribe the costs of the ingredients to a program.

A second limitation is that the effectiveness measure may not capture all the impacts resulting from onsite support. It is possible that onsite support affects other key outcomes not measured here. For example, schools receiving online support had higher attrition rates than schools receiving onsite support, suggesting that there may be other program benefits that accrue to onsite schools that keep them engaged in the program longer. If researchers can confirm that schools with missing data dropped out of the school health program (vs just not providing outcome data), then they might consider either (1) adjusting the overall costs by including the costs of schools that dropped out, or (2) obtaining outcome data from schools that did not complete the assessment annually. Either approach would likely make the program less cost‐effective, either by increasing the cost for a given level of effectiveness or by lowering the effectiveness for a given cost.

Based on our experience, we recommend that future researchers consider the implications of using self‐reported data. In this study, both baseline and outcome data were self‐reported by schools. To explore whether school‐reported outcome data was sufficiently reliable and to mitigate response bias, we interviewed a subset of participating schools that reported changes on a subset of assessment items. Although average levels of agreement between school data and independent interview data were found to be above 85%, schools often reported slightly higher scores than were justified, to reflect gains that would otherwise have gone undetected had they adhered strictly to the definitions of the 4‐point response scale. Future researchers might consider (1) triangulating self‐reported data with other data sources to examine reliability or (2) developing more sensitive measures of school health outcomes to account for schools' tendency to slightly inflate their self‐reported outcome data.

### Conclusions

By demonstrating the relative CE of 2 approaches for supporting implementation of a school health program, this study contributes to recent research showing that school wellness programs can play an important role in improving overall student wellness.^15^ The study also provides some initial evidence that providing online support and assistance for school health programs may be more cost‐effective than providing onsite support. Furthermore, online support is potentially even more cost‐effective when one considers the low incremental costs of scaling to more schools. Because the costs of providing online support are relatively fixed, regardless of the number of schools participating in the program—compared to onsite support, for which staffing costs increase proportionally to the number of schools supported, holding effectiveness constant—the online support CE increases as the number of schools receiving online support increases.

## IMPLICATIONS FOR SCHOOL HEALTH

The CE analysis of online versus onsite implementation support for a school health program suggests that online support is a promising way to promote healthy eating and physical activity in elementary schools that serve students from low‐SES households who are at high risk of obesity. Investments in online implementation support for school health programs may cost less than onsite support and produce similar improvements in students' health and reductions in childhood and adolescence obesity rates. However, online schools had higher attrition from the study than onsite schools over the 4 years. Because online implementation support may be more cost‐effective than onsite support but may not provide enough support to keep developing school health programs engaged, school staff should assess their level of capacity to implement a school health program when determining whether to participate in online or onsite support. In addition, school staff should consider participating in a school health program with a group of schools. Spreading onsite support across schools within a district or within close proximity may mitigate some of the costs of school attrition and provide opportunities for program providers to distribute resource‐intensive, in‐person supports to more schools without reducing the outcomes achieved.

### Human Subjects Approval Statement

Westat's institutional review board (IRB) is a specially constituted committee established to protect the rights and welfare of human participants. The IRB operates under procedures set forth in the regulations of the US Department of Health and Human Services and in the Federal‐wide Assurance granted to Westat by the Office for Human Research Protections. IRB approval is required before research may begin, continue, or be changed by the research team. Westat is committed to upholding regulatory and ethical standards through an FWA number, FWA00005551, issued by OHRP. Westat's IRB Organization number is IORG0000410, and Westat's IRB number is 00000695. The Westat IRB reviewed the Healthy Schools Randomized Trial, Project 6166, and determined the project is a program evaluation and exempt from IRB review on July 24, 2013.

### Conflict of Interest

The research team received funding from The JPB Foundation (Grant/award #207) for this study, which was part of the Healthy Schools Randomized Trial.
